# The Factors Affecting the Clinical Outcome and Integrity of Arthroscopically Repaired Rotator Cuff Tears of the Shoulder

**DOI:** 10.4055/cios.2009.1.2.96

**Published:** 2009-05-30

**Authors:** Nam Su Cho, Yong Girl Rhee

**Affiliations:** Department of Orthopaedic Surgery, Kyung Hee University School of Medicine, Seoul, Korea.

**Keywords:** Shoulder, Rotator cuff tear, Arthroscopic repair, Repair integrity, Retear

## Abstract

**Background:**

The purpose of this study was to evaluate the functional and anatomic results of arthroscopic rotator cuff repair, and to analyze the factors affecting the integrity of arthroscopically repaired rotator cuff tears of the shoulder.

**Methods:**

One hundred sixty-nine consecutive shoulders that underwent arthroscopic rotator cuff repair, had a postoperative MRI evaluation and were followed for at least two years were enrolled in this study. The mean age was 57.6 years (range, 38 to 74 years) and the mean follow-up period was 39 months (range, 24 to 83 months).

**Results:**

The rotator cuff was completely healed in 131 (77.5%) out of 169 shoulders and recurrent tears occurred in 38 shoulders (22.5%). At the last follow-up visit, the mean score for pain during motion was 1.53 (range, 0 to 4) in the completely healed group and 1.59 (range, 0 to 4) in the group with recurrent tears (*p* = 0.092). The average elevation strength was 7.87 kg (range, 4.96 to 11.62 kg) and 5.25 kg (range, 4.15 to 8.13 kg) and the mean University of California at Los Angeles score was 30.96 (range, 26 to 35) and 30.64 (range, 23 to 34), respectively (*p* < 0.001, *p* = 0.798). The complete healing rate was 87.8% in the group less than 50 years of age (49 shoulders), 79.4% in the group over 51 years but less than 60 years of age (68 shoulders), and 65.4% in the group over 61 years of age (52 shoulders, *p* = 0.049); it was 96.7% in the group with small-sized tears (30 shoulders), 87.3% in the group with medium-sized tears (71 shoulders), and 58.8% in the group with large-sized or massive tears (68 shoulders, *p* = 0.009). All of the rotator cuffs with a global fatty degeneration index of greater than two preoperatively had recurrent tears.

**Conclusions:**

Arthroscopic repair of full-thickness rotator cuff tears led to a relatively high rate of recurrent defects. However, the minimum two-year follow up demonstrated excellent pain relief and improvement in the ability to perform the activities of daily living, despite the structural failures. The factors affecting tendon healing were the patient's age, the size and extent of the tear, and the presence of fatty degeneration in the rotator cuff muscle.

Among various surgical treatment methods used for the repair of a rotator cuff tear,^1^-3) arthroscopic surgery is currently the preferred method. However, arthroscopic repair of a full-thickness rotator cuff tear poses technical challenges. The arthroscopic procedures in the developmental stage have been evaluated only for their short- and mid-term efficacy.^4^-6) With regard to the short-term follow-up studies, arthroscopic repair of the rotator cuff has been reported to have good outcomes,^4^,6) result in less postoperative pain, cause less damage to the deltoid muscle, and enable patients to undergo rehabilitation and recover more rapidly.^4^,6) Even though there are known advantages, the outcomes after arthroscopic surgery are not as well established as those after open procedures.^7^-11) In addition, recent studies have reported a high rate of structural failure after arthroscopic rotator cuff repair.12) Since prior reports have shown a direct correlation between the postoperative clinical outcome and anatomic healing of the rotator cuff,^9^,^13^-15) evaluation of anatomic healing, to assess the integrity of the rotator cuff repair, might be crucial to assessing the outcome of the arthroscopic repair. However, there is a paucity of information, in the literature, on the extent of anatomic healing after arthroscopic rotator cuff repair and the factors associated with this process.16)

The purpose of this study was to assess the functional outcomes according to anatomic healing observed with magnetic resonance imaging (MRI) after arthroscopic rotator cuff repair. In addition, we examined the factors associated with healing by comparing the clinical results between a group with complete healing and a group with recurrent tears. During the current study, we considered the following two hypotheses: as long as the loop and the knot was securely fixated intraoperatively, the anatomical healing after arthroscopic rotator cuff repair, should be similar to open or mini-open procedures; and the group with recurrent tears should have poorer clinical outcomes than the group that completely healed with regard to pain relief and the activities of daily living.

## METHODS

### Patient Selection

Among consecutive patients followed up from March 1997 to October 2005, 169 consecutive shoulders treated with arthroscopic repair for rotator cuff tear and available for at least 2 years of follow-up and postoperative MRI evaluation at a minimum of six months following surgery were enrolled in this study. Those patients with acromioclavicular arthritis that required distal clavicle resection, advanced glenohumeral arthritis, stiffness, or rotator cuff tears with a workers' compensation claim, or those patients that required a tenotomy or tenodesis of the long head of the biceps, were excluded from the study. Patients undergoing revision procedures were also excluded. The indication for surgery was the failure of a conservative treatment for a chronic tear over at least three months.

### Preoperative and Postoperative Evaluations

Examinations were made one day before operation and during the follow-up period. All the operative results were analyzed at the last follow-up. Preoperative and postoperative subjective pain at rest and during active shoulder motions was measured with the visual analogue scale (VAS). The VAS was used to measure pain of the patients with 0 indicating no pain and 10 indicating extremely severe pain. Active shoulder motions including forward flexion, external rotation at the side, internal rotation to the back and cross body adduction were measured by goniometer with the patient in the seated position preoperatively at 3-week, 6-week, 3-month, 6-month, 12-month postoperatively and at the last follow-up. Each measurement was repeated two times and the average value of measurements was recorded. Quantitative strength measurements of the rotator cuff were obtained with use of a portable, handheld Nottingham Mecmesin Myometer (Mecmesin Co., Nottingham, UK).^17^,18) Elevation strength was tested with the patient in the seated position with the arm flexed to 90 degrees in scapular plane. The Simple Shoulder Test,19) American Shoulder and Elbow Surgeons (ASES) score20) and the Shoulder Rating Scale of the University of California at Los Angeles (UCLA)21) were used for clinical assessment.

### Tear Classification

The extent of the tear was determined intraoperatively under direct arthroscopic visualization after debridement of the degenerated tendon edges. The tear size was measured in the anterior-posterior dimension using a calibrated probe introduced through the posterior portal while viewing from the lateral portal. According to the classification of DeOrio and Cofield,22) it was classified into small-sized, medium-sized, or large-sized and massive tear.

### Operative Techniques

All operations were performed by the senior author with the patient in a beach chair position with the back of the bed flexed about 70°. Both shoulders were examined under general anesthesia for the range of motion (ROM). A posterior portal was established for the initial assessment of the joint. An anterior portal through the rotator interval was established as the working portal for intra-articular debridement. Four portals were typically required for rotator cuff repair: posterior and posterolateral portals were used mainly for the standard 30° angled 4-mm arthroscope (the viewing portals), while anterosuperior and lateral portals were used for the instruments (the working portals). Except 41 cases of flat type of acromion on preoperative radiograph, young age of patient, and tear due to acute trauma, subacromial decompression with acromioplasty was performed in 128 cases (76%). After finishing subacromial decompression, the posterolateral portal was used as viewing portal for 'Grand Canyon' view23) and the posterior and anterosuperior portal were used as 'waiting room' portal.24) After adequate visualization, preparation, and release of the tendon, the upper surface of the greater tuberosity was abraded widely with a shaver, removing all soft tissue and cortical bone, to create a bleeding cancellous bone bed. The greater tuberosity was gently debrided and smoothed of irregularities and the superficial bone was decorticated, but the medullary canal was not exposed. A hole was punched in the greater tuberosity with the bone punch 45° to the horizontal plane through an accessory superolateral portal. The suture anchor, a 5-mm Super Revo (Linvatec, Largo, FL, USA) pre-threaded with two #2 braided polyester sutures (green and white), was inserted through the accessory superolateral portal. The rotator cuff repair was performed by first placing a suture shuttle through the tendon with use of a suture hook (Linvatec) or Banana SutureLasso (Arthrex, Naples, FL, USA). A suture-hook or Banana SutureLasso was inserted through the working portal or modified Neviaser portal and was used to pass the suture some distance medial to the tendon edge, close to the musculotendinous junction. The shuttle was used to bring one end of the suture through the tendon. An arthroscopic knot was tied, reducing the tendon to the bone. The suture anchors were used depending on tear size and configuration. The arm was immobilized in a sling following routine portal closure. Fifty-nine tears (35%) were repaired with use of a combination of side-to-side tendon sutures and tendon-to-bone repair (with anchors), and one hundred (59%) were repaired with use of suture anchors alone. An isolated side-to-side repair was performed over abraded bone in only ten patients (6%).

### Postoperative Rehabilitation

On the first postoperative day, the patients started passive ROM exercises, including pendulum exercises, passive forward flexion, and external rotation. They performed passive ROM exercises in a tolerable range at first and were instructed to do the exercises three times a day, 10 rounds each time. No active motion was allowed for six weeks or until passive motion had been completely recovered. Active-assisted motion was initiated at six weeks postoperatively. Then, muscle strengthening exercises followed. A return to recreational activity with heavy demands on the shoulder or to manual labor was delayed for six months.

### Assessment of Tendon Healing

In order to assess tendon healing, anatomic evaluation of the cuff repair was done with use of a MRI as the investigation of choice, as it provides the benefits of multiplanar imaging of the postoperative shoulder. A postoperative MRI performed at a minimum of six months following surgery. All studies were obtained with a 1.5-T unit (Signa, GE Medical Systems, Milwaukee, WI, USA) by using the routine pulse sequences. The images were reviewed by one experienced senior radiologist who was informed that the patients had undergone surgery for rotator cuff repair and blinded to the size and location of the tear that had been repaired. Continuity and rerupture of the tendon was assessed on MR images according to established MR imaging criteria.^25^,26) When a fluid-equivalent signal or nonvisualization of the supraspinatus, infraspinatus, or subscapularis tendon was found on at least one T2-weighted or proton-density-weighted image, the diagnosis of a full-thickness retear, anatomic failures of healing, was made. The extent of the retear was determined in the coronal plane, according to a modification of the system described by Thomazeau et al.15) If the tear edge was lying over the greater tuberosity, then the minor retraction was classified as a stage-I retear (usually < 1 cm in greatest diameter). If the tear exposed the humeral head but did not retract to the glenoid articular surface, then medium retraction was considered to be a stage-II retear (between 1 and 3 cm in greatest diameter). Tears that extended to the glenoid were considered to have large retraction and were classified as a stage-III retear (between 3 and 5 cm in greatest diameter). Tears that were severely retracted medial to the glenoid were classified as a stage-IV retear (> 5 cm in greatest diameter). The presence of fatty degeneration was evaluated for each muscle using the five stage grading system developed by Goutallier et al.27) A global fatty degeneration index (GFDI) was calculated for each shoulder as the mean value of three muscles.

### Statistical Analysis

The Wilcoxon signed rank test was performed to assess the difference in preoperative and postoperative range of motions and shoulder scores. The Mann-Whitney U test was used to compare the results between complete healing group and retear group. Pearson's chi-square test was used to analyze the factors affecting the healing of repaired rotator cuff tears. The SPSS ver. 12.0 (SPSS Inc., Chicago, IL, USA) was used for all statistical analyses, with the α level set at 0.05.

## RESULTS

### Preoperative Patient Demographics

The mean age at the time of operation was 57.6 years (range, 38 to 74 years) and the mean follow-up period was 39 months (range, 24 to 83 months). There were 101 males and 68 females. A total of 129 patients had surgery on the dominant shoulder, and 40 on the non-dominant shoulder. Fifty-five shoulders (32.5%) had a history of trauma. The average duration of symptoms before surgery was 15 months (range, 1 to 60 months). The arthroscopic findings showed small-sized tears in 30 shoulders (17.8%), medium-sized tears in 71 (42%), and large-sized and massive tears in 68 (40.2%).

### Pain

The VAS score for pain at rest decreased from the preoperative mean of 1.55 (range, 0 to 4) to 0.26 (range, 0 to 3) at the last follow-up visit. The VAS for pain during exercise reduced remarkably to 1.56 (range, 0 to 4) from the preoperative mean of 6.24 (range, 3 to 10). The improvement of pain both at rest and during exercise was statistically significant (*p* = 0.016, < 0.001) (Table 1).

### Muscle Strength and Shoulder Motion

The elevation strengths improved significantly from the preoperative mean of 4.39 kg (range, 1.82 to 8.16 kg) to 6.56 kg (range, 4.15 to 11.62 kg) at the last follow-up visit (*p* < 0.001). The mean preoperative range of active motion was 155° (range, 80 to 175°) of forward flexion and 49° (range, 10 to 90°) of external rotation. At the last follow-up visit, the mean range of motion improved to 164° (range, 135 to 180°) of forward flexion, and 53° (range, 35 to 90°) of external rotation. The postoperative improvement of forward flexion and external rotation was statistically significant (*p* = 0.002, 0.007).

### Clinical Assessments

The mean ASES score improved from 40.2 points (range, 8.3 to 78 points) preoperatively to 91.7 points (range, 55 to 100 points) at the last follow-up visit (*p* < 0.001). The mean UCLA score increased from 18.5 points (range, 6 to 26 points) preoperatively to 32.4 points (range, 22 to 35 points) at the last follow-up visit (*p* < 0.001), with 87 shoulders (51.5%) rated as excellent, 73 (43.2%) as good, and 9 (5.3%) as poor. The mean simple shoulder score rose from 5.3 (range, 1 to 8) preoperatively to 10.9 (range, 8 to 12) at the last follow-up visit (*p* < 0.001). During the ≥ 2 years of postoperative follow-up, the patients were asked to fill out a questionnaire designed to assess their satisfaction with the surgery on a scale of 0 to 100, and the average satisfaction score was 90.4.

### Anatomic Results

The rotator cuff was completely healed in 131 (77.5%) of the total 169 shoulders, and had a recurrent tear in 38 shoulders (22.5%). The size of the cuff recurrent tear was classified as stage I in 18 shoulders (47%), stage II in 17 shoulders (45%), and stage III in 3 shoulders (8%). The average age at the time of surgery was significantly younger (*p* = 0.016) in the complete healing group with 53.2 years (range, 38 to 67 years), while it was 58.4 years (range, 45 to 74 years) in the group with recurrent tears. The VAS score at rest at the last follow-up visit was an average of 0.25 (range, 0 to 3) and 0.27 (range, 0 to 3), respectively. The VAS score during motion averaged 1.53 (range, 0 to 4) in the group with complete healing, slightly superior to the 1.59 (range, 0 to 4) in the group with recurrent tears; however, these differences were not statistically significant (*p* = 0.248, 0.092). At the last follow-up visit, the average elevation strength was significantly higher (*p* < 0.001) in the group with complete healing, 7.87 kg (range, 4.96 to 11.62 kg) than the 5.25 kg (range, 4.15 to 8.13 kg) in the group with recurrent tears. With regard to the range of active motion, forward flexion was an average of 165° (range, 140 to 180°) and external rotation was a mean of 53° (range, 35 to 90°) in the group with complete healing, while it was 163° (range, 135 to 180°) and 52° (range, 35 to 85°), respectively in the group with recurrent tears. Although the average ROM was greater in the group with complete healing, this difference did not reach statistical significance (*p* = 0.256, 0.416). The ASES score at the last follow-up in-creased to 92.1 points (range, 58 to 100 points) in the group with complete healing and to 91.3 points (range, 55 to 100 points) in the group with recurrent tears. The improvement was also noted in the UCLA score with 32.6 points (range, 24 to 35 points) for the group with complete healing and 32.2 points (range, 22 to 35 points) for the group with recurrent tears. The ASES score and the UCLA score were significantly improved postoperatively; however, these changes were not statistically significant (*p* = 0.407, 0.798). With regard to the average patient satisfaction, the score was 90.6 for the group with complete healing and 90.2 points for the group with recurrent tears; these findings indicated good results but they did not reach statistical significance (*p* = 0.940) (Table 2).

### Factors Associated with Healing of the Tendon

#### Age

For the assessment of tendon healing, the patients were subdivided into three groups according to age: those ≤ 50 years, 51 ≤ and ≤ 60 years, and ≥ 61 years of age. Complete healing was observed in 43 (87.8%) out of 49 shoulders ≤ 50 years, in 54 (79.4%) out of 68 shoulders 51 ≤ and ≤ 60 years, and in 34 (65.4%) out of 52 shoulders ≥ 61 years. The incidence of rotator cuff recurrent tears tended to increase with age at the time of surgery (*p* = 0.049) (Table 3).

#### Tear size

Tears were classified into small-sized, medium-sized, and large-sized and massive ones according to the measurements performed during surgery. Complete healing was observed in 29 (96.7%) out of 30 small tears, in 62 (87.3%) out of 71 medium tears, and in 40 (58.5%) out of 68 large and massive tears. It appeared that the smaller the intraoperative tear size, the higher the rate of complete healing; this finding reached statistical significance (*p* = 0.009) (Table 4).

#### Preoperative fatty degeneration of cuff muscles

Complete healing was found in 92.5% of shoulders with a global fatty degeneration index of ≤ 0.25, in 88.3% of shoulders with an index of 0.25 ≤ and ≤ 1, in 52.9% of shoulders with an index 1 ≤ and ≤ 1.5, and in 38.5% of shoulders with an index 1.5 ≤ and ≤ 2. The recurrence of tears was observed in all nine shoulders with an index ≥ 2. When the severity of the fatty degeneration of the cuff muscles was higher preoperatively, there was a greater chance of a recurrent tear; this difference was statistically significant (*p* = 0.014) (Table 5).

#### Duration of symptoms before surgery

The patients were divided into two groups depending on whether the duration of symptoms before surgery was less or more than one year. Complete healing was observed in 75 (83.3%) out of the 90 shoulders that underwent surgery within one year of the onset of symptoms, and in 56 (70.9%) out of 79 shoulders that were treated more than one year after the onset of symptoms. Although there was a trend towards higher rates of complete healing when the duration of symptoms before surgery was less than one year, these findings did not reach statistical significance (*p* = 0.215).

#### Trauma

The patients were divided into two groups depending on the history of trauma. Of 55 shoulders who had been diagnosed as rotator cuff tear for shoulder pain caused directly by trauma, 45 (81.8%) were completely healed after the arthroscopic rotator cuff repair. Among 114 shoulders that had no history of trauma, 86 (75.4%) had complete healing. Therefore, a history of trauma before surgery did not appear to affect tendon healing (*p* = 0.983).

#### Operative technique

Complete healing was observed in 83 (82.2%) out of 101 shoulders where only suture anchors were used, in 44 (74.6%) out of 59 shoulders in whom a combination of side-to-side tendon sutures and suture anchors were employed, and in 4 (44.4%) out of 9 shoulders whose tears were repaired with side-to-side tendon sutures only. The results for the repairs involving only suture anchors and those using additional side-to-side tendon sutures were similar (*p* = 0.942).

## DISCUSSION

Recurrent tears after rotator cuff repair is a common and well-known problem^9^,^13^,^15^,28) usually diagnosed by ultrasonography,^9^,13) arthrography,7) or CT.27) Since thomazeau et al.15) and Knudsen et al.28) used MRI for the assessment of rotator cuff repair, MRI has been accepted as the most useful examination tool.25) It has advantages over ultra-sonography and arthrography in terms of sensitivity and specificity, especially for the assessment of full-thickness tears.26) In the present study, the anatomical evaluation of the cuff repair was done with MRI as the investigation of choice for the assessment of tendon healing of the shoulder postoperatively.

Wolf and Bayliss11) reported that 16 (70%) out of 23 arthroscopically treated rotator cuff tears were observed to be normal in second-look arthroscopy performed at an average of five months postoperatively. Gleyze et al.8) reported that tendon healing was noted in 61% of the patients after arthroscopic repair of supraspinatus tendon tears. Wilson et al.10) reported complete healing with second-look arthroscopy in 22 (67%) out of 33 patients that had arthroscopic staple fixation. Boileau et al.16) reported relatively good results with arthroscopic supraspinatus tendon repair; there was complete healing of the tendon in 46 (71%) out of 65 shoulders at least six months postoperatively. By contrast, Galatz et al.12) reported high recurrence rates, 17 (94%) out of 18 cases, based on observations using sonography at least 12 months after arthroscopic repair of supraspinatus tendon tears ≥ 2 cm. In this current study, we found a 22.5% recurrence rate similar to other studies on arthroscopic repair, and comparable failure rates of 20-54%^7^,^9^,^13^,^28^,29) reported in studies on open rotator cuff repairs. Many biomechanical studies30) have shown that arthroscopic rotator cuff repairs using suture anchors produce relatively higher recurrence rates than open or mini-open repair procedures involving bone tunnels because of the low pressure distribution and insufficient contact. However, based on our study results, we could confirm our hypothesis that with a secure loop and knot fixation, arthroscopic rotator cuff repair results in similar anatomic tendon healing rates compared to open or mini-open repairs.

According to prior studies, better clinical results tend to occur in completely healed tendons after surgery.^9^,^13^-15) Therefore, it is important to maintain the integrity of the repaired rotator cuff until complete healing is achieved between the tendon and the tuberosity. Even so, considering that good clinical results could often be observed in recurrent tears of rotator cuffs, it is confusing to associate the integrity of the rotator cuff repair with the final outcome. In the absence of adequate information, establishing their association would improve our understanding of the pathophysiology of the rotator cuff. Jost et al.14) investigated 20 cases of recurrent tears following open repair, and found significant clinical improvement compared to the preoperative status in spite of structural failure. Galatz et al.12) reported that although 17 patients had recurrent tears after arthroscopic repair, and good results could be identified in 13 (72%) among them with regard to the ASES score (≥ 90), pain relief, recovery of ROM, activities of daily living, and muscle strength. Harryman et al.9) and Gazielly et al.13) showed that completely healed rotator cuffs were more functional than the ones with recurrent tears after an open repair; however, Liu and Baker29) reported that no association could be found at the last follow-up visit between the functional outcome and the preservation of the repaired rotator cuff following arthroscopically assisted mini-open repairs. In the current study, with regard to pain relief and the recovery of ROM, although the group with complete healing had good results compared to the group with recurrent tears, the differences were not statistically significant. With regard to the ASES score, the UCLA score, and patient satisfaction, no significant difference was found between the two groups. In other words, in spite of the structural failure, the clinical results of the group with recurrent tears after arthroscopic repair were comparable to those with completely healed rotator cuffs in terms of pain relief and the recovery of range of active motion.

Comparisons of the changes between the preoperative and the postoperative muscle strength in patients with recurrent tears have been the focus of studies by only a small number of investigators. Jost et al.14) reported that even partial healing led to significant improvements in abduction after surgery. Boileau et al.16) reported that although functional improvements could be expected from patients with either partial healing or recurrent tears, the muscle strength was lower than that of patients with complete healing. The results of our study were consistent with the findings of Boileau et al.16); the muscle strength at the last follow-up visit was significantly higher in the group with complete healing compared to the group with recurrent tears.

With regard to the factors associated with complete healing after rotator cuff repair, Boileau et al.16) reported that as the age at the time of surgery was older and the tear was larger and more extensive, the rate of tendon healing was lower. However, gender, duration of symptoms and injection therapy prior to surgery, secondary gain from compensation, or the employment of acromioplasty had no association with tendon healing. Harryman et al.9) followed 105 patients with repaired rotator cuffs by an open method for an average of five years; assessment of tendon healing showed the following: complete healing was found in 80% of the cases with a supraspinatus tendon tear alone; recurrent tears were found in 43% of the cases with supraspinatus tendon tears accompanied by infraspinatus tendon tears; recurrent tears were observed in over two thirds of the cases with supraspinatus, infraspinatus, and subscapularis tendon tears. Gazielly et al.13) reported that as the size of the initial tears was smaller, the healing rate was greater, based on their observations made after open repair; while recurrent tears were found in 7% of the shoulders with a supraspinatus tendon tear, they were noted in 41% of the shoulders with both supraspinatus and infraspinatus tendon tears. They concluded there was a strong correlation between the size of the initial tear and recurrent tears. In our study, as the age at the time of surgery was older, the complete healing rate was lower: in the ≤ 50 age group, the rate was 87.8% and in the ≥ 61 age group, it was 65.4%. In addition, it appeared that there was a correlation between the tear size and the rate of complete healing, and this finding reached statistical significance: the rate was 96.7% in patients with small tears and 58.8% in patients with large or massive tears. However, the duration of symptoms before surgery, trauma, and the operative technique did not appear to have a significant influence on tendon healing. Goutallier et al.18) analyzed 220 cases of rotator cuff repair and found recurrent tears in 79 cases (36%). They suggested that patients with posterosuperior tears were more vulnerable to recurrent tears. Particularly, with regard to the impact of the preoperative fatty degeneration of the cuff muscles on the anatomic and clinical results, recurrent tears were observed more often in patients with a global fatty degeneration index of > 1; the Constant score at the last follow-up visit was low, even in patients with complete healing, if the preoperative fatty degeneration index was high. In this study, recurrent tears were found in more than half of the shoulders with preoperative global fatty degeneration index scores of ≥ 1. The findings suggest that as the extent of the preoperative fatty degeneration was greater, the rate of complete healing was lower. Therefore, the presence of fatty degeneration is a major prognostic factor for the outcome of rotator cuff repairs.

In conclusion, arthroscopic repair of full-thickness rotator cuff tears had a relatively high rate of recurrent defects. However, the minimum two-year follow up demonstrated excellent pain relief and improvement in the ability to perform the activities of daily living despite the structural failures, except with regard to elevation strength. The factors affecting tendon healing included the patient's age at the time of operation, the size and extent of the tear, and the presence of fatty degeneration in the rotator cuff muscle.

## Figures and Tables

**Table 1:**
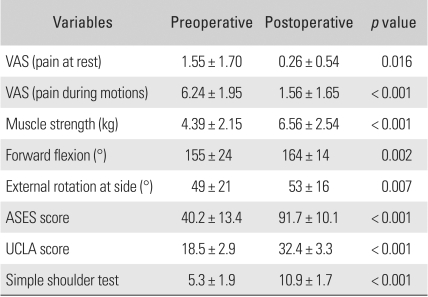
Clinical Outcomes of Arthroscopic Rotator Cuff Repair

VAS: Visual analogue scale, ASES: American shoulder and elbow surgeons, UCLA: University of California at Los Angeles.

**Table 2:**
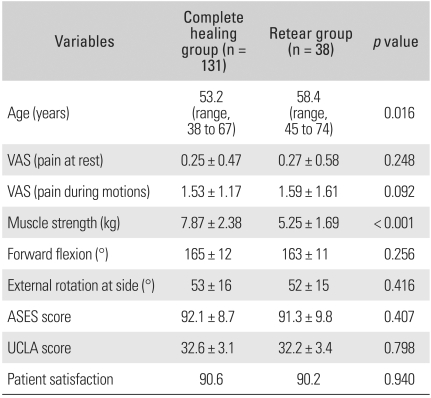
Anatomic Results of Arthroscopic Rotator Cuff Repair

VAS: Visual analogue scale, ASES: American shoulder and elbow surgeons, UCLA: University of California at Los Angeles.

**Table 3:**
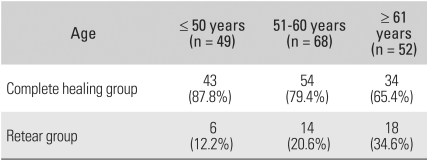
The Prevalence of Tendon Healing according to Age

**Table 4:**
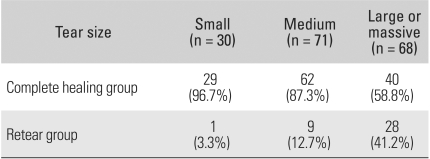
The Prevalence of Tendon Healing according to Preoperative Tear Size

**Table 5:**
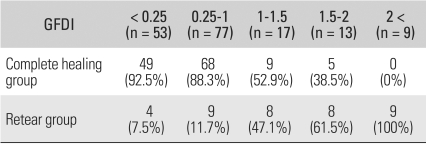
The Prevalence of Tendon Healing according to Preoperative Cuff Muscle Fatty Degeneration

Criteria for grading muscle fatty degeneration: Grade 0: No fatty deposits; Grade 1: Some fatty streaks, Grade 2: More muscle than fat, Grade 3: As much muscle as fat, Grade 4: Less muscle than fat. For each shoulder, we evaluated fatty degeneration, not only in each cuff muscle individually but in all cuff muscles combined, by calculating the global fatty degeneration index (GFDI) as the mean value of the grades for the supraspinatus, infraspinatus, and subscapularis.
